# Differentiation-dependent chromosomal organization changes in normal myogenic cells are absent in rhabdomyosarcoma cells

**DOI:** 10.3389/fcell.2023.1293891

**Published:** 2023-11-07

**Authors:** Joe Ibarra, Tyler Hershenhouse, Luay Almassalha, David Walterhouse, Vadim Backman, Kyle L. MacQuarrie

**Affiliations:** ^1^ Department of Pediatrics, Feinberg School of Medicine, Northwestern University and Ann & Robert H. Lurie Children’s Hospital of Chicago, Chicago, IL, United States; ^2^ Department of Gastroenterology and Hepatology, Northwestern Memorial Hospital, Northwestern University, Chicago, IL, United States; ^3^ Department of Biomedical Engineering, Northwestern University, Evanston, IL, United States

**Keywords:** myogenesis, rhabdomyosarcoma, differentiation, chromosome, nuclear organization

## Abstract

Myogenesis, the progression of proliferating skeletal myoblasts to terminally differentiated myotubes, regulates thousands of target genes. Uninterrupted linear arrays of such genes are differentially associated with specific chromosomes, suggesting chromosome specific regulatory roles in myogenesis. Rhabdomyosarcoma (RMS), a tumor of skeletal muscle, shares common features with normal muscle cells. We hypothesized that RMS and myogenic cells possess differences in chromosomal organization related to myogenic gene arrangement. We compared the organizational characteristics of chromosomes 2 and 18, chosen for their difference in myogenic gene arrangement, in cultured RMS cell lines and normal myoblasts and myotubes. We found chromosome-specific differences in organization during normal myogenesis, with increased area occupied and a shift in peripheral localization specifically for chromosome 2. Most strikingly, we found a differentiation-dependent difference in positioning of chromosome 2 relative to the nuclear axis, with preferential positioning along the major nuclear axis present only in myotubes. RMS cells demonstrated no preference for such axial positioning, but induced differentiation through transfection of the pro-myogenic miRNA miR-206 resulted in an increase of major axial positioning of chromosome 2. Our findings identify both a differentiation-dependent, chromosome-specific change in organization in normal myogenesis, and highlight the role of chromosomal spatial organization in myogenic differentiation.

## Introduction

Myogenesis, the development of skeletal muscle, is a tightly regulated and coordinated sequential process that results in cells proceeding from the state of proliferating myoblast to terminally differentiated myotube (Bentzinger et al., 2012). Through the feed-forward action of a small number of myogenic regulatory factors (Penn et al., 2004; Berkes and Tapscott, 2005; Cao et al., 2006), hundreds of genes are significantly differentially regulated during the process (MacQuarrie et al., 2013; Choi et al., 2020), ultimately resulting in the final post-mitotic, multi-nucleated form of myotubes. These large-scale expression changes have been shown to be associated with changes in the organization of nuclear space at such levels as regions of the genome (Robson et al., 2016; Doynova et al., 2017), the positioning of specific chromosomal areas (Neems et al., 2016), and the association between centromeres and nucleoli (Rodrigues et al., 2023). Some of these changes have been demonstrated to play causative roles in the regulation of involved genes (Neems et al., 2016; Robson et al., 2016), demonstrating the importance of changes in the organization of the components of the nucleus to the process of myogenesis as a whole.

Rhabdomyosarcoma (RMS) is a tumor of skeletal muscle, and the most common of the pediatric soft tissue sarcomas (Rudzinski et al., 2021). RMS possess many similarities to normal myogenic cells: from morphology, to gene expression of the myogenic regulatory factors (Tapscott et al., 1993; Yang et al., 2009), to the genome-wide binding patterns of myogenic regulatory factors to DNA (MacQuarrie et al., 2013). Despite those similarities, RMS cells suffer from a differentiation defect that permits them to continue to proliferate, unless induced to differentiate by various mechanisms (Taulli et al., 2009; Yang et al., 2009; Rao et al., 2010; Macquarrie et al., 2012; MacQuarrie et al., 2013), many of which affect components of the normal myogenic pathway. RMS is also notable for the fact that some tumors bear a characteristic PAX-FOXO transcription factor gene fusion, while others do not (McEvoy et al., 2023). Gene fusion status impacts not only multiple facets of tumor cell biology (Begum et al., 2005; Davicioni et al., 2006; Cao et al., 2010; Gryder et al., 2017; Vicente-Garcia et al., 2017; Hanna et al., 2018; Boudjadi et al., 2021; Sunkel et al., 2021; Zhang et al., 2022), but clinical outcomes as well (Hibbitts et al., 2019). Despite their similarity to normal myogenic cells, less is known about what deficits may or may not be present in RMS at the level of higher-order organization, such as chromosomal positioning and genomic organization.

In this study, we utilized cultured cells to investigate the organizational characteristics of two chromosomes–chromosome 2, which has been shown to be enriched for tandem gene arrays (TGAs) of myogenic genes, and chromosome 18, which is not enriched for myogenic TGAs (Neems et al., 2016). By comparing the characteristics of those chromosomes in multiple RMS cell lines, both those with and without the characteristic PAX-FOXO gene fusion, to the characteristics seen in both proliferating and differentiated normal myocytes, we sought to 1) characterize the extent of similarities and differences in chromosomal organization both between the tumor cell lines and tumor and normal cells and 2) identify potential chromosomal level organizational deficits in RMS cells. We found that, while both chromosomes occupied greater nuclear area in all tumor cell lines compared to normal cells, the positioning of chromosome 2 relative to the nuclear periphery was largely preserved in the tumor cells. In contrast, chromosome 18 exhibited a greater frequency of occupying the more peripheral area of the nucleus in tumor compared to normal cells. More strikingly, we found a chromosome- and differentiation-specific difference in positioning relative to the nuclear axis. In differentiated myotubes, chromosome 2 was found positioned close to the major axis of the cell far more frequently than would be expected by chance, while the positioning in myoblasts and RMS cells did not show this predilection. In contrast, chromosome 18 positioning was indistinguishable from what would be expected by chance in all cell types and conditions. Induced differentiation in the RMS cells increased the frequency of chromosome 2 positioning along the major axis of the cell, suggesting a relationship between chromosomal spatial positioning and the regulation of myogenic genes in the tumor cells.

## Materials and methods

### Cell culture

Cell lines were obtained from ATCC and experiments were performed in cells at low passage number. RD and SMS-CTR cells were cultured and maintained in DMEM media (Gibco) supplemented with 10% fetal bovine serum and 1% PS (penicillin-streptomycin) (Gibco). RH30 cells were cultured and maintained in RPMI (Gibco) with 10% fetal bovine serum and 1% PS. Primary human myoblasts were cultured and maintained in Mesenchymal stem cell media (ATCC) with the addition of the contents of the primary human skeletal muscle growth kit (ATCC) and 1% PS. Myotubes were generated by having myoblasts reach confluency, and then cultured for 96 h in differentiation tool media (ATCC) with the addition of 1% PS. For transfected cells, they were plated onto poly-L-lysine coated coverslips which were prepared by submerging glass coverslips in poly-L-lysine solution (MilliporeSigma) for 15 min at 37° before rinsing three times with PBS, and then placing under UV light for 15 min prior to use.

### Transfections

One day prior to transfection, cells were trypsinized in 0.05% trypsin-EDTA and plated onto poly-L-lysine coated glass coverslips (see above) at sufficient density to reach 50%–60% confluency the following day. Cells were transfected using either a miR-206 miRNA mimetic (Thermofisher) or negative control mimetic #1 (Thermofisher) using RNAiMax (Thermofisher). Transfections were performed according to manufacturer’s protocol with the following modifications: the mimetic’s final concentration was 7 nM, no antibiotics were present in the media, and 1.5 uL of RNAiMax was used per well for a 12 well dish. After 24 h, cells were washed 2x with PBS, and media was changed to low serum differentiation media (DMEM +1% horse serum (Hyclone) + 1x insulin-transferrin-selenium (Corning) + 1% PS). Cells were fixed using 4% paraformaldehyde (PFA) (Electron Microscopy Sciences) in phosphate-buffered saline (PBS) after 48 h in differentiation media.

For fluorescent oligo transfections, the Block-iT Alexa Fluor Red Fluorescent Oligo (Invitrogen) was transfected at the concentration indicated using RNAiMax as above, with the following modifications. Cells were kept light-protected as much as possible, and 24 h after transfection, cells were fixed as above, mounted with Diamond antifade with DAPI (Invitrogen), and then visualized using microscopy. Cells were counted manually for positivity for oligo signal and nuclei number.

### Chromosome paint hybridization for adherent cells

Cells were trypsinized, resuspended in appropriate growth media and then plated onto glass coverslips and allowed to adhere for approximately 16 h prior to fixation. Cells were rinsed once in PBS prior to fixation in 4% PFA for 10 min at room temperature. Cells were then washed 3 times in PBS, followed by incubation in PBS with 0.01% Triton X-100 at room temperature 3 times for 3 min, then incubation in 0.5% Triton X-100/1x PBS at room temperature for 15 min. Cells were then incubated in 20% glycerol/PBS at room temperature for 3–4 h. Cells were washed in PBS 3 times for 10 min each and then incubated in 0.1 N HCl for 5 min at room temperature. The cells were incubated in 2x SSC twice for 3 min each before being placed in 50% formamide (Electron Microscopy Sciences)/2x SSC at room temperature for approximately 18 h. After the addition of chromosome paints (Metasystems) to the coverslips, slides were heated to 75°C for 2 min before being placed at 37° for approximately 72 h for hybridization. After hybridization, coverslips were washed in 2x SSC washes at 37° three times for 5 min each, followed by washes in 0.1x SSC at 60° three times for 5 min each. The coverslips were then washed in 4x SSC/0.2% Tween-20 three times for 3 min and mounted on microscope slides using Diamond antifade with DAPI (Invitrogen).

### Image acquisition and analysis

Images were acquired on a Nikon Confocal Microscope or a Zeiss LSM 800 Confocal microscope. Imaging was done with a Plan Apo VC 100 × 1.4 NA oil objective as a multidimensional z-stack. The acquired 3D image stacks were then fed through imaging processing pipelines utilizing standard tools on Cell Profiler and Fiji (Schindelin et al., 2012; Stirling et al., 2021). The pipelines performed the functions of translating images to maximal projections, calculating distance, object size, nuclear size and eccentricity, object intensity as a function of nuclear concentric rings, area occupation, object intensity distribution, angle theta, and the centroid of an object.

#### Three-dimensional reconstructions

For 3D reconstruction images, cell images were analyzed with the ZEN blue edition software (version 3.5.093.00008, Carl Zeiss Microscopy GmbH). Z-Stacks were obtained using standard software tools, and 3-D images were rendered by adjusting the brightness within the linear range to allow for optimal chromosome visualization.

#### Axial orientation determination

Using standard Mathematica tools (Wolfram Research, 2022), a script was developed to organize and analyze collected data (https://github.com/luaypurple/Chromosome-Spatial-Analysis). In summary, the orientation of chromosomes along the major and minor axis of their parent cell was assessed by calculating the angles of each chromosome relative to the nuclear center and classifying them based on their orientation. Chromosomal orientation was classified based on the calculated angle between the centroid of the identified chromosomal signal and the determined nuclear axes. Chromosome centroids located between 45° and 135° relative to an axis were grouped separately from those with an angle less than or equal to 45° and greater than or equal to 135°.

Chromosome orientation along the major and minor axis was assessed in two ways: 1) as a summation of all chromosomes analyzed and 2) on a per cell basis. Myoblasts and myotubes were split into two classifications: 1) both chromosomes along the minor axis and 2) one or both chromosomes along the major axis. RMS cells were classified into two groups: 1) greater than or equal to 50% of chromosomes on the major axis, 2) fewer than 50% of chromosomes on the major axis.

#### Nuclear and chromosomal DAPI intensity

To approximate DNA density in chromosome 2 and chromosome 18 regions, we utilized the confocal max-projections obtained as described above with both Chromosome-Paint and DAPI staining. Nuclear borders were defined by DAPI intensity using the in-built Mathematica function FindThreshold utilizing Kapur’s Method of entropy minimization. To account for spatial variations in staining intensity prior to nuclear-mask segmentation, the inbuilt Non-localMeansFilter function was applied to the DAPI intensity images, normalizing staining over a 1-pixel radius to produce mask boundaries. Composite images with a 2-pixel dilation and 2-pixel erosion were then produced to account for the effect of the DAPI intensity normalization. The resulting masks were then produced and defined as nuclear areas of interest. Areas by this process were excluded if they were greater than 10,000 pixel or if they extended beyond the image boundary. Noting that despite this restriction, multiple adjacent nuclei in close proximity (e.g. multinucleated cells) could still potentially be considered as one nuclear area of interest. Chromosomal masks were produced using Threshold with piecewise garrot partitioning paired with Kapur’s Method, restricting analysis to chromosomes with areas occupying between 100 and 10,000 pixel. To account for Chromosome-Paint staining variation, we utilized a median filter over a 2-pixel radius. As non-biological effects influence staining intensity (e.g. variability in magnification, illumination intensity, and acquisition time), we restricted our analysis to the ratio of DAPI intensity within a chromosome region of interest compared to the DAPI intensity of the accompanying whole nucleus. Using the inbuilt function, ComponentMeasurements, median DAPI intensity within chromosome regions of interest and accompanying nuclear regions could be obtained. We finally calculated the relative DAPI intensity within the chromosome to the nucleus as follows:.

$$R_{fragment}=\frac{Median\;Chromosome\;DAPI}{Median\;Nuclear\;DAPI}$$

#### Statistical analysis

**FIGURE 1:**
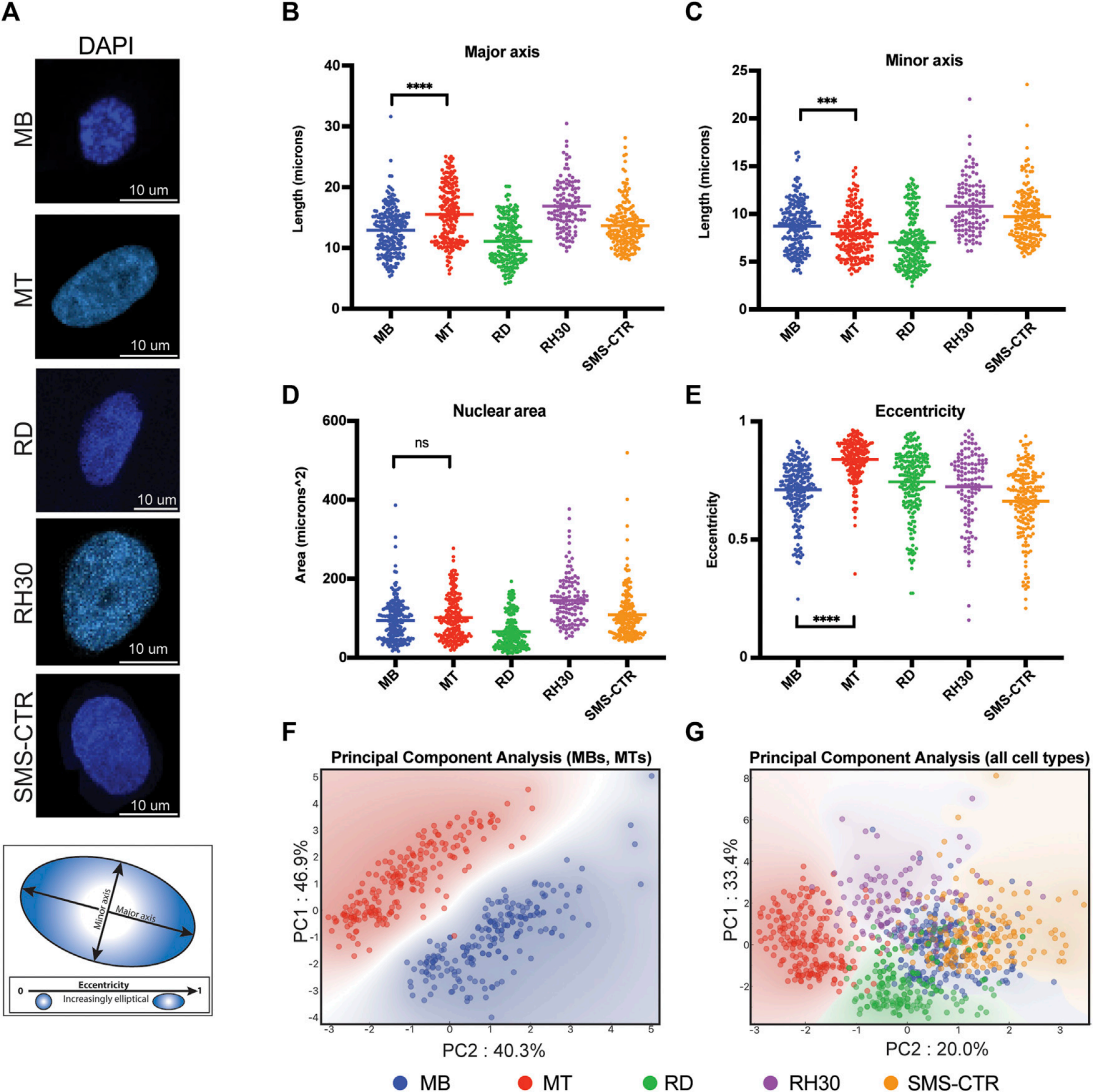
Nuclear shape and size of rhabdomyosarcoma cell lines are more similar to myoblasts than myotubes. **(A)** Representative microscopy images of the DAPI-labeled nuclei of primary human myocytes and rhabdomyosarcoma cell lines. Scale bar as indicated. Diagram below shows representative major and minor axes in a nucleus. **(B)** Scatterplot demonstrating the major axis length of each of the indicated cell types. Each point indicates the measurement from a single nucleus, with the mean indicated by the horizontal line. Only the statistical testing between MB and MT cells is indicated. **(C)** Scatterplot demonstrating minor axis length measurements as in **(B)**. **(D)** Scatterplot demonstrating area measurements, as for **(B)**. **(E)** Eccentricity measurement scatterplot, as for **(B)**. **(F)** Principal component analysis was performed using the measurements from B-E for myoblasts (MB) and myotubes (MT) only, and demonstrates a strong separation between the cell types based only on those nuclear characteristics. Each axis label (PC1 and PC2) indicates the respective percentage of variability explained. **(G)** When the three rhabdomyosarcoma cell lines are included in the PCA, overlap is seen between the myoblasts (MB) and rhabdomyosarcoma cells, but not the myotubes (MT) and rhabdomyosarcoma cells. As for 1F, the respective percentage of variability that is explained is listed on each axis. Statistical testing done by t-tests with unequal variance. ns: not significant; ***: *p* < 0.001; ****: *p* < 0.0001, n = 212 (MB), 199 (MT), 214 (RD), 121 (RH30), 181 (SMS) nuclei.

**FIGURE 2:**
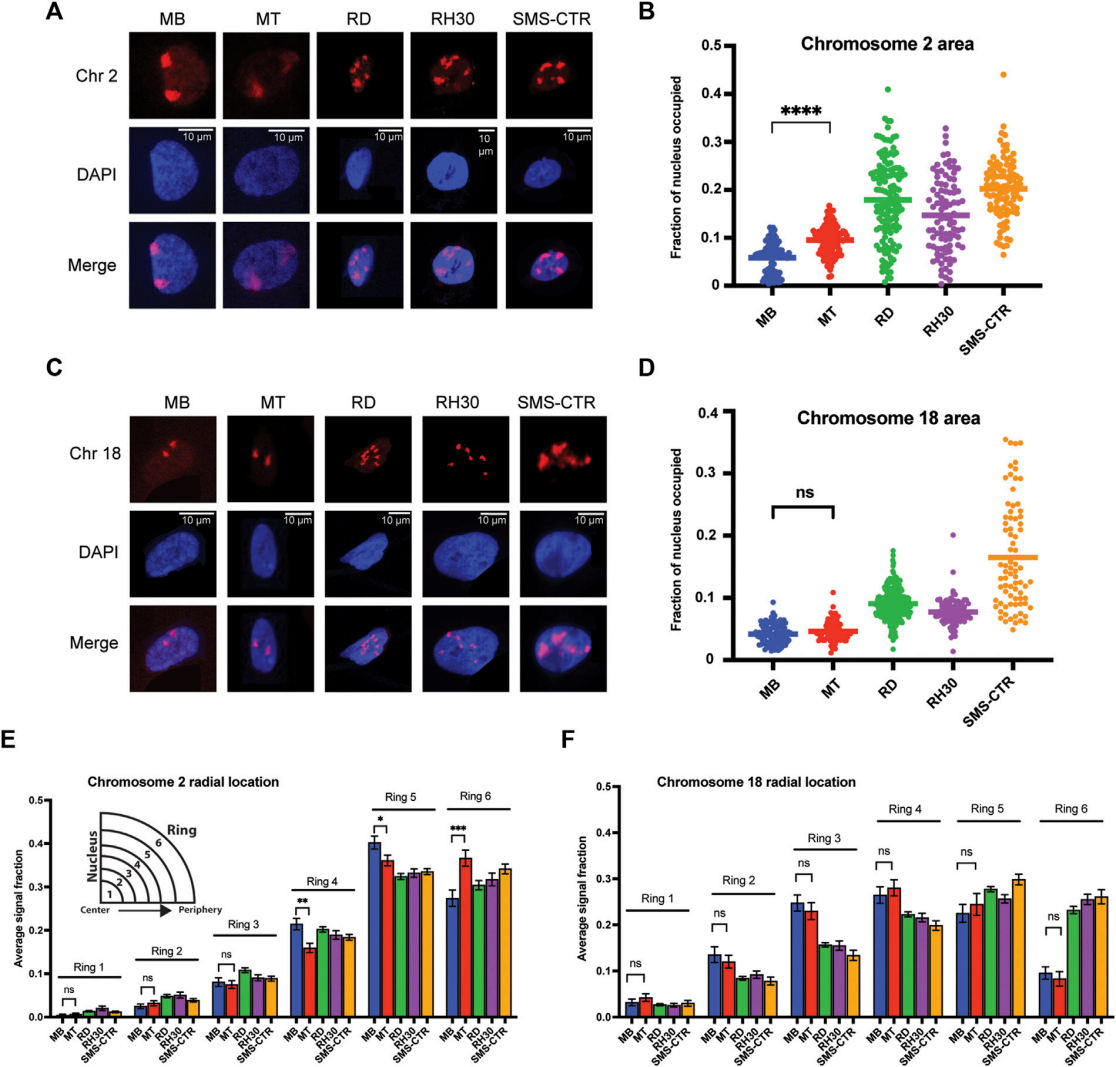
Chromosome-specific differences in organizational characteristics with differentiation and in tumor cells. **(A)** Representative microscope images of myoblasts (MB), myotubes (MT) and rhabdomyosarcoma cell lines with chromosome 2 visualized through the hybridization of a chromosome paint. Images represent visualization of either the paint fluorophore (Chr 2), the nucleus (DAPI), or the merge of the above, as indicated to the side. **(B)** The area of the nucleus occupied by chromosome 2 was determined for each cell type, and is represented as a scatterplot, with each nucleus measured represented as a single point, and the mean represented by the horizontal bar. Statistical testing is shown only for the MB to MT comparison and demonstrates a significant increase in area occupied by chromosome 2 in the differentiated cells. **(C)** Representative microscope images for chromosome 18 visualization as in 2A. **(D)** Plots of the area of the nucleus occupied for chromosome 18, as in 2B. Statistical testing of the means of MBs and MTs demonstrated no significant difference in area occupied. **(E)** Graphing of the average fraction and SEM of chromosome 2 fluorescent signal present in each one of six concentric nuclear rings (ring 1: most central, ring 6: most peripheral) demonstrates that in all cell types analyzed, chromosome 2 had little presence in the inner rings compared to the outer rings, and that there was a shift to increased signal presence in the outermost ring with differentiation from MB to MT. **(F)** Graphing, as in 2E, of the average fraction of chromosome 18 fluorescent signal in each of six concentric nuclear rings demonstrates more central localization compared to chromosome 2, and no difference in radial localization with the process of differentiation. ns: not significant; *: *p* < 0.05; **: *p* < 0.01; ***: *p* < 0.001; ****: *p* < 0.0001. n = 100–143 nuclei per cell type for chromosome 2; 78–270 nuclei for chromosome 18.

**FIGURE 3:**
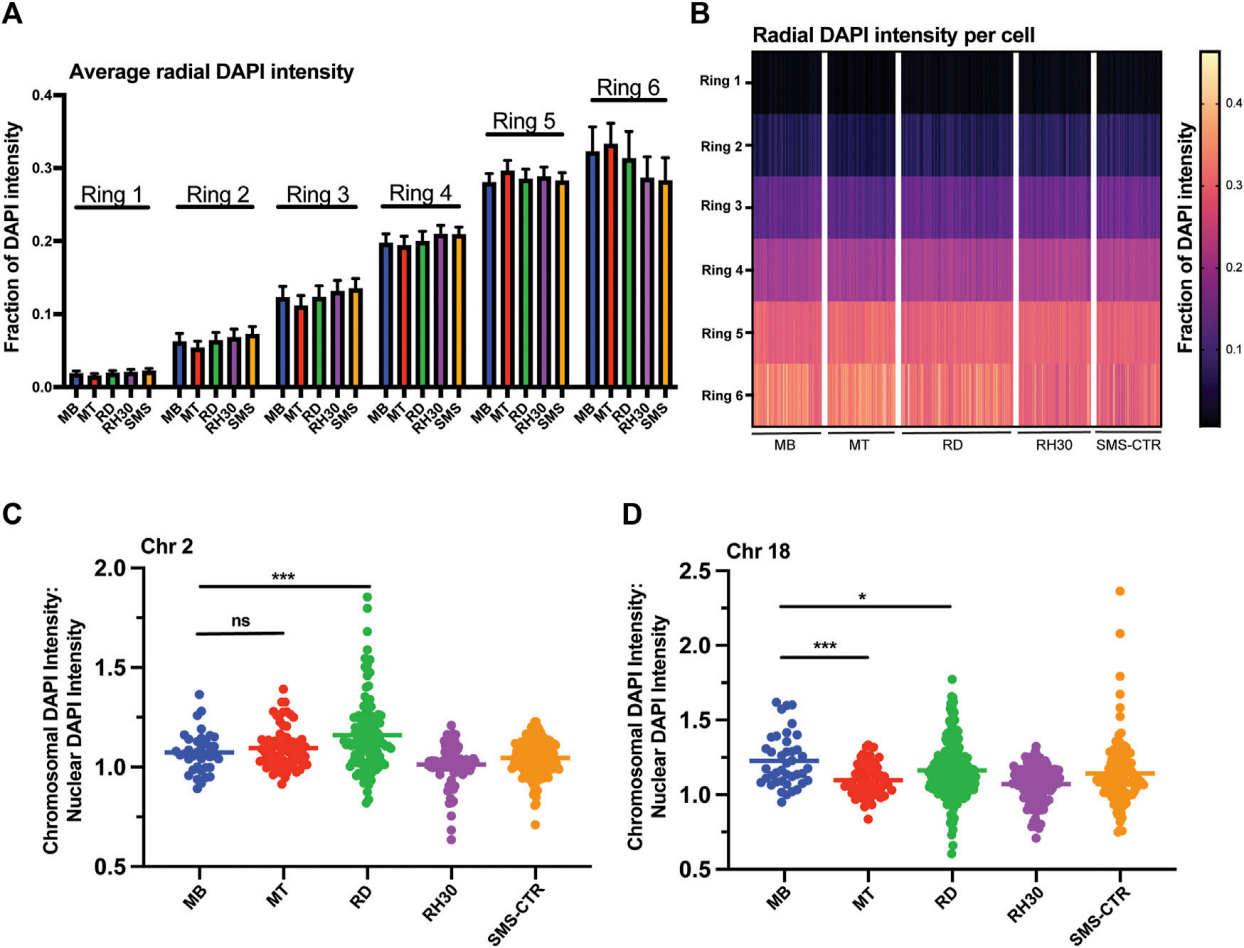
Myogenic and RMS cells exhibit similar overall DNA density patterning, but cell- and chromosome-specific differences in chromosome DNA density. **(A)** DAPI intensity in nuclei as a function of radial distance is shown divided up across six concentric nuclear rings, where ring 1 is the innermost portion of the nucleus and ring 6 the most peripheral. Each bar represents the average intensity for a given cell type in a given ring, with error bars indicating the standard deviation. **(B)** The data from 3A shown here as a heat map, with each column representing a single individual nucleus. **(C)** A scatterplot depicting the ratio of the DAPI intensity of the region of the nucleus occupied by chromosome 2 relative to the normalized DAPI intensity of the whole nucleus for each cell type as indicated. Each point represents a single chromosome, the mean is indicated by the horizontal line. **(D)** A scatterplot depicting chromosome 18-specific DAPI intensity relative to normalized nuclear DAPI intensity as in 3C. Statistical testing done by t-tests with unequal variance. ns: not significant; *: *p* < 0.05; ***: *p* < 0.001; n = 96–167 nuclei per cell type for A and B; n = 37–167 chromosomes per cell type for chromosome 2; 41–228 chromosomes for chromosome 18 for C and D.

**FIGURE 4:**
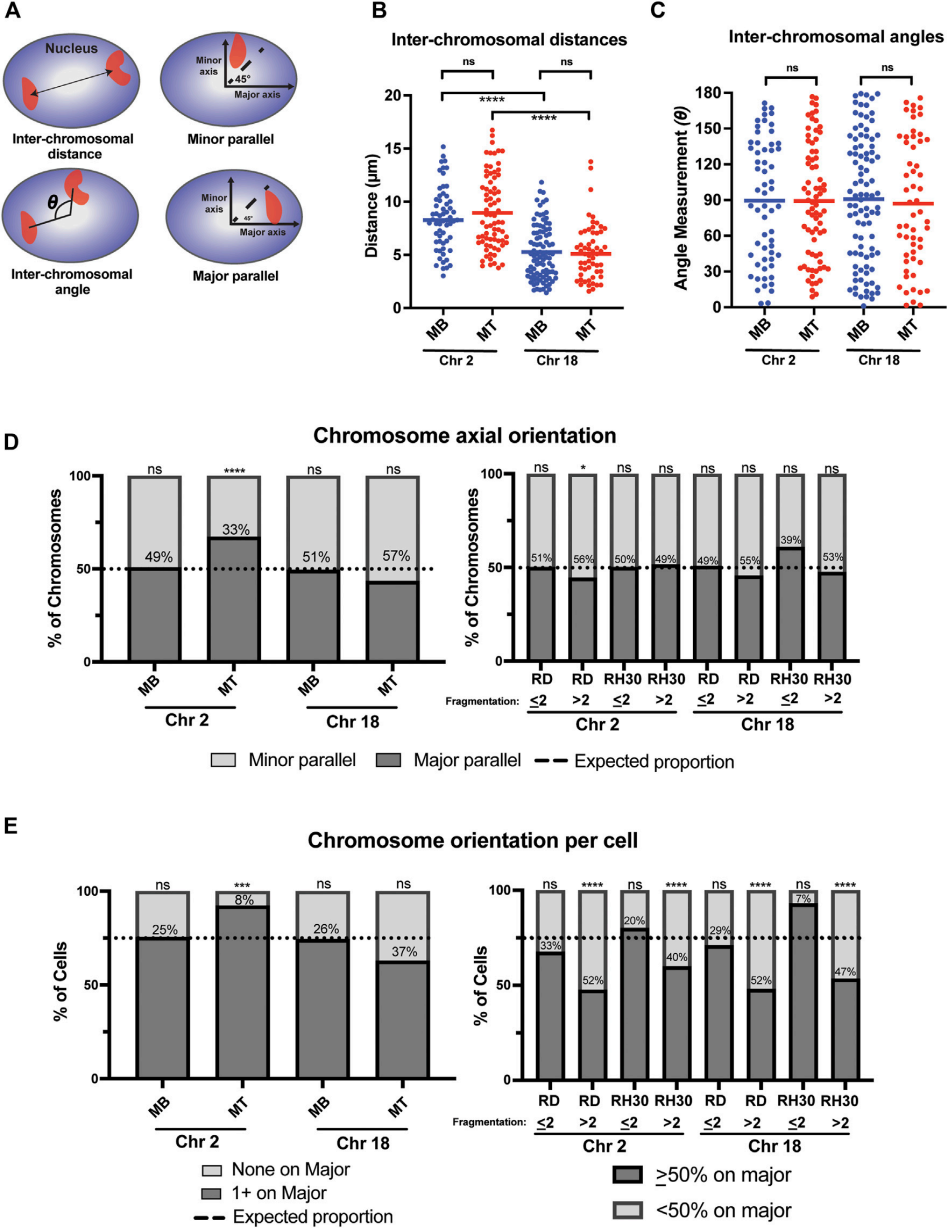
A chromosome-specific change in positioning relative to the nuclear axis occurs with normal myogenic differentiation and is absent in rhabdomyosarcoma cells. **(A)** Diagrammatic depictions of inter-chromosomal distance, inter-chromosomal angle, and minor and major parallel of chromosomal signal. **(B)** A scatterplot representing the inter-chromosomal distance between the centroids of either chromosome 2 or chromosome 18 (indicated below) in the nuclei of either myoblasts (MB) or myotubes (MT). Each point represents the measurement in a single nucleus, and the horizontal line represents the mean. **(C)** The scatterplot shows the inter-chromosomal angle for the same nuclei and chromosomes as measured in 4B. **(D)** Bar graphs that indicate the percentage of all measured chromosomes for an indicated cell type that were assigned either as being associated with the minor axis (minor parallel) or the major axis (major parallel), as depicted in 4A. The dotted line indicates the expected proportion of chromosomes that would be found associated with a given axis if they were organized in space at random. **(E)** Bar graphs for the same cells and measurements as in 4D, but with the calculation done as the orientation of the chromosomes in each individual nucleus. Nuclei in normal cells were classified either as having no chromosomes assigned to the major axis (none on major) or at least 1 chromosome assigned to the major axis (1 + on major). In tumor cells, cells were grouped as either having <50% of their identified chromosomal signals on the major axis, or ≥50% of chromosomal signals on the major axis. As in 4D, the dotted line indicates where the expected division would be between the two classifications if each chromosome was randomly positioned in nuclear space. In both D and E, the number within the bars indicates the value assigned to the minor parallel for each condition. Statistical testing in B and C was by t-tests with unequal variance; D and E was by using a binomial test to compare observed count frequencies. ns: not significant; *: *p* < 0.05; ***: *p* < 0.001; ****: *p* < 0.0001; n = 55–93 nuclei per cell type: chromosome pairing in B and C; n = 80–140 nuclei per cell type for chromosome 2 in D and E; n = 60–220 nuclei per cell type for chromosome 18 in D and E.

**FIGURE 5:**
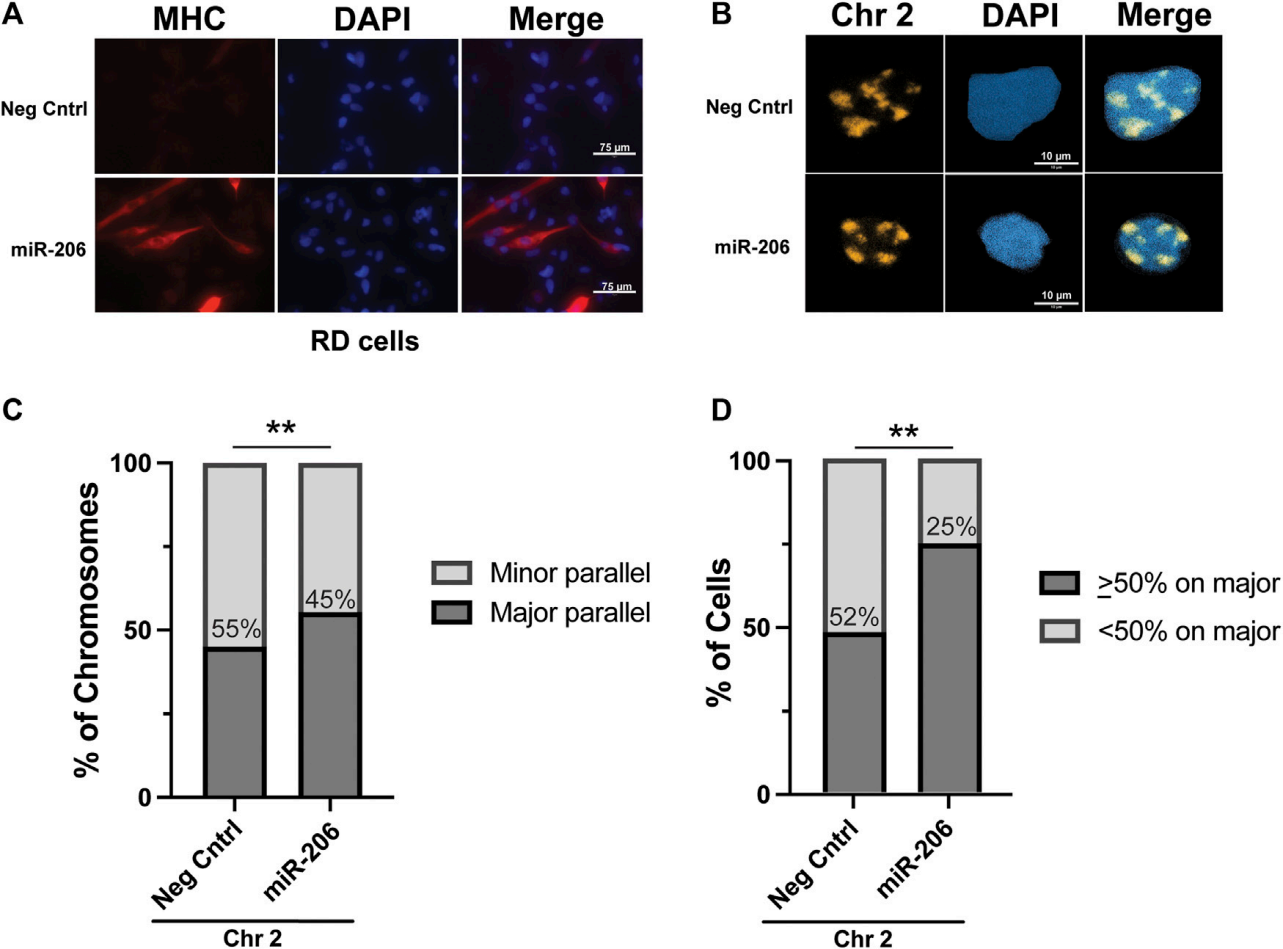
Differentiation of RD cells via transfection of a miRNA miR-206 mimetic increases the frequency of chromosome 2 positioning along the major nuclear axis. **(A)** Immunohistochemistry for myosin heavy chain, a marker of myogenesis, shows an increase in its expression and the formation of elongated cellular shapes, some of which exhibit multi-nucleation, both consistent with differentiation of RMS cells specifically in those cells transfected with the miR-206 mimetic and not the negative control mimetic. **(B)** Representative images of chromosome 2 visualization via hybridization of chromosome paint in RD cells. **(C)** The proportion of chromosome 2 assigned as being associated either with the minor axis (minor parallel) or the major axis (major parallel) in RD cells transfected as indicated. The number within the bars indicates the value assigned to the minor parallel for each condition. **(D)** The proportion of cells with chromosome 2 positioning as indicated on a per-cell basis. The percentage of cells with <50% of chromosome signals along the major axis is indicated within the bars. For both 5C and 5D, statistical testing was performed using Fisher’s exact test for proportions. **: *p* < 0.01. n = 76 and 61 nuclei for negative control and miR-206 nuclei, respectively.

Statistical testing was performed using Prism 9. Pairwise comparisons were calculated on datasets consisting of, at a minimum, biologically independent duplicate samples using two-tailed t-test with Welch’s correction so as not to assume equal standard deviations in Figures 1–4 and Supplemental Figure S4. The regression in Supplemental Figure S2 was performed using the non-linear regression tool in Prism 9 and the least squares calculation for the regression. The observed *versus* expected calculations in Figure 4 used the binomial test in Prism 9 for comparing observed to expected count frequencies. The pairwise comparisons in Figure 5 were performed using Fisher’s exact test in Prism 9. Heat maps were generated using Prism 9’s function. Principal component analysis was performed using Orange3 (Demsar et al., 2013).

## Results

### Rhabdomyosarcoma cells exhibit similar patterns of nuclear shape and size characteristics as human myoblasts

To assess differences in nuclear characteristics between normal human myogenic cells and rhabdomyosarcoma (RMS) cells, cultured proliferating human myoblasts (MB), differentiated myotubes (MT) and cell culture models of RMS representing both PAX-FOXO fusion-negative (RD, SMS-CTR) and fusion-positive (RH30) subtypes had their nuclei visualized using DAPI staining (Figure 1A). Given all cell lines were grown in adherent conditions and consistently exhibited relatively little depth (typically ∼3–4 μm, data not shown) their characteristics were considered as projections into only the x- and y-axes, rather than as three-dimensional structures.

Nuclear characteristics including length of major and minor axes (Figures 1B,C), nuclear area (Figure 1D) and eccentricity (Figure 1E) were determined for each cell type. Differentiation of myoblasts into myotubes resulted in an average increase of major axis length from 12.8 to 15.5 μm (Figure 1B), an average decrease of minor axis length from 8.7 to 7.9 μm (Figure 1C), no difference in nuclear area (Figure 1D), and an average increase in eccentricity from 0.71 to 0.84 (Figure 1E). Principal component analysis (PCA) of the normal myogenic cells demonstrated that these basic nuclear characteristics were sufficient to distinguish proliferating myoblasts from differentiated myotubes with a high degree of fidelity and account for 87% of the variability in the data (Figure 1F).

While RMS cell lines typically exhibited similar distributions as the normal cells for their nuclear characteristics, as demonstrated by comparable coefficients of variation for individual characteristics, (Supplemental Table S1) their average values frequently differed both between tumor cell lines (Figures 1B,C, consider RD *versus* RH30) and when tumor cells were compared to the normal cells (Figure 1C, consider RD relative to MB and MT). The characteristic that all RMS cell lines demonstrated a fairly consistent pattern with was eccentricity, where they universally exhibited lower eccentricity compared to human myotubes (Figure 1E). PCA analysis that included both RMS cells and the normal myogenic cells demonstrated that, while the variability that was accounted for decreased to 53%, all RMS cell lines showed overlap with the proliferating MBs, while differentiated MTs demonstrated separate clustering (Figure 1G).

### Chromosomes 2 and 18 exhibit distinct patterns of nuclear area occupancy and radial localization in myogenic and RMS cells

A subset of human chromosomes have previously been identified as having a greater number of myogenic tandem gene arrays (TGAs)–comprised of linear stretches of genes differentially regulated during the process of myogenesis (Neems et al., 2016)–than would be expected by chance. We chose to interrogate the differences in a variety of chromosome organizational characteristics in normal myogenic and RMS cells in a chromosome with a significant enrichment for myogenic TGAs (Chromosome 2) and a chromosome without such enrichment (Chromosome 18). Chromosome 2 is approximately three times as long linearly as Chromosome 18 (∼242 million bp compared to ∼76 million bp). Re-analysis of the published data identifying myogenic TGA enrichment identified the presence of both up- and downregulated genes on both chromosomes: 420 genes differentially regulated by myogenesis on chromosome 2 (163 downregulated, 257 upregulated), and 106 genes differentially regulated genes on chromosome 18 (34 downregulated, 72 upregulated).

For both chromosomes, the same cell lines examined in Figure 1 were hybridized with chromosome paints to allow their visualization alongside DAPI staining to delineate their nuclei (Figures 2A,C). In all cell lines, the chromosomes typically occupied all or the majority of the z-axis (Supplemental Figure S1) so, as for Figure 1, imaging was projected into two dimensions prior to analysis. Imaging analysis universally identified two chromosome signals per nucleus for the myogenic cells, while variable numbers were identified in tumor cells (Figures 2A,C and Supplemental Figure S2A,B), consistent with chromosomal fragmentation and/or aneuploidy. All the tumor cell lines used have previously been described as being, at minimum, hyperdiploid (Hinson et al., 2013).

The area of the nucleus occupied by chromosome signal showed differences both in normal myogenic cells, as well as between normal and tumor cells. While nuclear area occupancy of chromosome 2 nearly doubled on average as myogenic cells differentiated (MB: 5.9%, MT: 9.5% of nuclear area occupied), the area occupied by chromosome 2 in the tumor cells was variable from cell to cell, but with notably higher averages than the normal cells (Figure 2B). In contrast, chromosome 18 showed no difference in nuclear area occupied during normal myogenesis (Figure 2D, compare MB to MT), and while all tumor cell lines again demonstrated higher averages, for 2 of the 3 lines, the clustering around the mean was notably more consistent than for the last cell line (Figure 2D, compare RD/RH30 to SMS-CTR). For each chromosome, area occupied relative to the extent of chromosomal fragmentation was plotted, and showed no evidence of a consistent relationship (Supplemental Figure S2A,B), suggesting that the difference seen in the tumor cells is not a function of fragmentation and/or aneuploidy.

In addition to the area occupied, the radial positioning of each chromosome was determined as a function of the chromosome signal seen in each of six concentric rings per nucleus. Chromosome 2 exhibited very low average presence in the innermost nuclear rings (rings 1 and 2), moderate presence in the middle rings (3 and 4) and the most presence in the outermost rings (rings 5 and 6) (Figure 2E). Myogenic differentiation resulted in chromosome signal changes in rings 4-6, with a decrease in signal in both rings 4 and 5 (from 0.21 to 0.16 and 0.40 to 0.36, respectively), with a concomitant increase in signal in ring 6 (from 0.27 to 0.37), consistent with chromosome 2 shifting to a more peripheral localization with differentiation (Figure 2E, compare MB and MT bars). In contrast, while chromosome 18 also demonstrated extremely low occupancy in ring 1, it was seen more frequently in ring 2 and both the middle rings compared to chromosome 2, with no change in response to differentiation (Figure 2F).

When considering the radial localization in the tumor cell lines, notable differences were seen between the two chromosomes. Chromosome 2 localization did not clearly align more consistently with either myoblasts or myotubes (Figure 2E, consider ring 5, where all tumor lines are lower than both myogenic cell types and ring 4, where all tumor lines are instead intermediate between the myogenic cells), but all tumor cell lines demonstrated localization that was relatively comparable to that seen in the normal cells (Figure 2E). In contrast, chromosome 18 often exhibited substantial discrepancy between normal and tumor cells. Chromosome 18 signal was notably lower in rings 2-4 in all tumor cells compared to normal, and much higher in ring 6 compared to the normal cells (Figure 2F), consistent with a shift to more peripheral localization in the tumor cells. When the radial localization of each chromosome was visualized on a per-cell basis, rather than the cell-type averaging as above, despite cell-to-cell variability for each cell type, the same overall patterns as described above were clearly visible (Supplemental Figure S3A,B).

In summary, in normal myogenesis, the chromosome enriched for myogenic TGAs, chromosome 2, exhibits an increase in the nuclear area it occupies and a shift to a more peripheral localization with differentiation, while the non-enriched chromosome 18 changes neither its area nor its peripheral localization. In tumor cell lines, both chromosomes exhibit an increase in area relative to normal cells, but while chromosome 2 appears to maintain a fairly preserved radial occupancy compared to the normal cells, chromosome 18 has a substantial change in its positioning in all tumor lines tested when compared to normal.

### Chromosome specific changes in radial positioning and occupied area are not related to changes in local DNA density

Given the changes seen in radial positioning in normal cells for chromosome 2, and between normal and tumor cells for chromosome 18, we sought to determine if there was a detectable change in the positioning of either chromosome relative to areas of differing DNA density that could potentially correspond to large-scale regions of hetero- or euchromatin. Using DAPI intensity as a marker for DNA density, we sought to determine both whether large-scale variations in DNA density were observed, and whether there were differences in the overlap between chromosomal signal and DNA density between normal and tumor cells.

The average DAPI intensity was first calculated for each cell type as a function of the same six concentric nuclear rings used to determine radial chromosome localization. Regardless of the cell type analyzed, increasing DAPI intensity was consistently observed as concentric rings became more peripheral (Figure 3A). To account for the possibility that the tumor cells may possess significant cell-to-cell variability, but relatively consistent averages when compared to normal cells, the relative DAPI intensity in each ring was plotted for each individual nucleus and demonstrated a pattern consistent with that seen for the average value analysis, with increases in intensity across the innermost and middle rings, and highest intensity in rings 5 and 6, relatively comparable between the two (Figure 3B).

To investigate the relationship between chromosomal positioning relative to DNA density, the ratio of the DAPI intensity of the region of the nucleus occupied by a given chromosome to the normalized DAPI intensity of the nucleus as a whole was determined and plotted for both chromosomes 2 and 18 (Figures 3C,D). Chromosome 2 exhibited no significant difference comparing MBs to MTs (means of 1.07 and 1.1, respectively) while the RMS lines exhibited variable ratios, with RDs exhibited higher average values compared to MBs (mean of 1.16), consistent with chromosome 2 in those cells occupying areas of greater DAPI intensity, RH30s exhibiting lower average values compared to MBs (mean of 1.01) and SMS-CTRs were statistically indistinguishable from MBs (mean of 1.05). Chromosome 18, in contrast, did exhibit a difference between MBs and MTs, with chromosome 18 in MTs occupying areas of lower DAPI intensity on average, as did all tumor cell lines (Figure 3D).

### Myogenic cells exhibit differentiation-dependent preferential positioning of chromosome 2 along the major nuclear axis that is absent in rhabdomyosarcoma cells

Since myogenesis affects the inter-allelic distance of the two alleles of the myogenic transcription factor myogenin (Neems et al., 2016), we investigated whether there were differentiation dependent differences in inter-chromosomal distances and angles (Figure 4A). While chromosomes 2 and 18 exhibited chromosome-specific patterns in average inter-chromosomal distances, with both chromosomes 18 frequently being located more closely together than chromosomes 2, for a given chromosome no difference in average distance was seen between myoblasts and myotubes (Figure 4B). Similarly, no difference was noted in the inter-chromosomal angles with differentiation (Figure 4C), suggesting that there is no coordinated regulation of the positioning of either chromosome relative to each other with differentiation.

In contrast however, a chromosome-specific, differentiation-dependent difference was seen when assessing chromosomal positioning relative to the nuclear axis. When considering where chromosomes were located relative to the major and minor axis of the nucleus (Figure 4A), chromosome 2 in myoblasts showed positioning that was indistinguishable from what would be expected by chance (Figure 4D, leftmost bar), even if taking into account corrections for the average greater length of the major axis relative to the minor axis in those cells (Supplemental Table S2). In contrast, in myotubes, chromosome 2 was significantly more frequently identified along the major axis than would be expected to occur by chance, despite the even greater average major axis length in those cells compared to myoblasts (Figure 4D, consider Chr 2 MT bar). This relationship was also seen when chromosome positioning relative to the nuclear axis was considered on a per-cell basis (Figure 4E, left panel), with <10% of myotube nuclei having both chromosomes 2 located along the minor axis, less than half the percentage that would be expected by chance if chromosomes were randomly and independently distributed in nuclear space. This relationship continued to be statistically significant, even when accounting for axial length and area discrepancies (Supplemental Table S2). Chromosome 18, on the other hand, whether analyzed on a per-cell basis or for all the chromosomes as a whole, was found at proportions that were statistically indistinguishable from what would be expected by chance in both myoblasts and myotubes (Figures 4D,E, left panels).

Given the complexity of computing expected probabilities for tumor cells with fragmented chromosomes, the tumor nuclei were dichotomized into two groups for the axial analysis: those cells with two (or fewer) detectable chromosomal signals per nucleus and those with greater than 2 detectable chromosomal signals. Analysis of the distribution on the per chromosome basis showed only one condition that was significantly different from the distribution that would be expected by chance, with high fragmentation score RD cells having a slightly increased rate of chromosome 2 being aligned along the minor parallel (Figure 4D, right panel). When considering the per-cell distributions, results were consistently related to the fragmentation score: all chromosome-cell type pairs with a fragmentation score of ≤2 demonstrated a distribution indistinguishable from the expected proportion, while all chromosome-cell type pairs with a fragmentation score of >2 were significantly different from that proportion (Figure 4E, right panel). Overall, the distribution of positioning in high fragmentation cells demonstrated a relatively even distribution between major and minor axis positioning (Figure 4E, right panel, consider RD > 2 Chr 2 and RD > 2 Chr 18).

### Induction of differentiation in RD cells increases positioning of chromosome 2 along the major nuclear axis

With the RMS cell lines exhibiting patterns of chromosome 2 axial positioning similar to what was seen in myoblasts, as opposed to the preferential positioning seen in myotubes, we tested whether we could restore chromosome 2 to major axis positioning via the induction of differentiation in RMS cells. The introduction of the pro-myogenic miRNA miR-206 has previously been shown to lead to differentiation and cell cycle withdrawal in RMS cells(Taulli et al., 2009; Rao et al., 2010; Macquarrie et al., 2012). RD cells were transiently transfected (Supplemental Figure S4A) with either a miRNA mimetic of miR-206 or a negative control mimetic. As expected, incubation of transfected cells in low-serum differentiation media led to morphologic change and significant expression of the marker of myogenesis, myosin heavy chain (MHC), in cells transfected with the miR-206 mimetic but not the negative control mimetic (Figure 5A). Visualization of chromosome 2 in transfected cells worked as it had for untreated cells (Figure 5B), and the axial orientation of chromosomes was assessed on both a per chromosome (Figure 5C) and per cell (Figure 5D) basis. In both analyses, a statistically significant increase in the proportion of chromosomes along the major axis was found overall in those cells transfected with the miR-206 mimetic compared to those transfected with the negative control mimetic, with the per chromosome proportion increasing from 45% to 55% and the per cell proportion increasing from 48% to 75% (*p* = 0.0089 and 0.0024, respectively). Analysis based on fragmentation status was performed and showed no clear difference in proportions, though it was limited due to a small number of cells that met criteria for low fragmentation status, especially in the miR-206 mimetic condition (data not shown).

The nuclear characteristics of the transfected cells were assessed, to determine if miR-206 treatment would result in nuclei taking on myotube nuclei characteristics. There was no evidence of difference in the nuclear minor axis length, but major axis, area, and eccentricity all decreased on average with differentiation. For both of the axial length measurements and for the area, miR-206 transfection led to more homogeneity between nuclei, as represented by reduced coefficients of variation (Supplemental Figure S4B–E). There was a small but statistically significant shift to more internal localization of chromosome 2 in the miR-206 transfected cells, with a 2% decrease in the average signal in the outermost ring (6), and increases of only approximately 1% in rings 1–3 (Supplemental Figure S4F).

## Discussion

Previous work examining the role of specific chromosomes in myogenic cells has demonstrated that a subset of chromosomes are enriched for tandem gene arrays (TGAs) of genes that are differentially regulated during normal myogenesis. We now demonstrate that differential behavior in chromosome organization and spatial positioning occurs between a chromosome enriched for such TGAs (Chromosome 2) and one that is not enriched for them (Chromosome 18) during normal myogenesis. Most strikingly, we identify a preferential spatial positioning of chromosome 2 along the major nuclear axis in differentiated myotubes that is absent in both proliferating myoblasts, and baseline RMS cells, but can be partially restored in RMS cells that are induced to differentiate.

To obtain a basis for analyzing chromosomal topologies between normal myocytes and RMS cells, we characterized their nuclei on a variety of features, including axial measurements, area, and eccentricity. Others have demonstrated changes in myocyte nuclear volume and thickness with normal myogenesis (Rozwadowska et al., 2013; Kolanowski et al., 2020), with myotubes more ‘flattened’ and elongated with differentiation. While our analysis in two dimensions demonstrates no change in nuclear area, myotubes exhibit an increase in one dimension accompanied by a concomitant decrease in the other and an increased eccentricity, consistent with the previously described changes. The RMS cells exhibit notable variability both in the absolute values of their nuclear characteristics, and their relative relationship to the normal cell measurements. For all three RMS cell lines, their eccentricity was closer to myoblasts than myotubes, demonstrating a more rounded shape. Principal component analysis demonstrated that those four nuclear characteristics alone allowed for excellent discrimination of the undifferentiated from differentiated myocytes, and clustered the RMS cells with myoblasts, and distinctly apart from myotubes.

Interestingly, our induced differentiation of the RMS cells with the microRNA miR-206 led to changes in nuclear characteristics, but rather than the RD cells becoming more elliptical and elongated with differentiation as the normal myocytes did, they instead became smaller and more rounded. It is unclear if that is because 1) despite differentiation, the RMS cells do not (or cannot) appropriately regulate their nuclear morphology and size, 2) the changes in nuclear characteristics would ultimately occur if the RMS cells were kept in differentiation-promoting conditions for long enough, or 3) the fact that the transfected RMS cells were cultured on poly-L-lysine coated surfaces to tolerate subsequent hybridization steps without excessive cell loss. The poly-L-lysine did result in some alteration of nuclear size characteristics at baseline, as control transfected RD cells possessed somewhat difference mean nuclear axes measurements compared to non-transfected RD cells. Notably, while the range of eccentricity measurements was equivalent between control and miR-206 transfected cells, all three of the nuclear size characteristics became more homogeneous with induced differentiation. While the presence of the microRNA itself could not be directly tracked in individual cells, the increased homogeneity of the entire population of nuclei in the miR-206 condition agrees with the high efficiency of transfection of RD cells we saw as measured by transfection of a fluorescently labeled oligo. This in turn suggests that the expression of markers of myogenesis, such as myosin heavy chain, are separate - either temporally or in regards to regulation–from the processes that reshape the cell nucleus, as myosin heavy chain expression was not seen as widely in the miR-206 transfected cells as the changes in nuclear morphology were. Comparison to the induction of differentiation in RMS cells using other tools, such as MEK inhibition (Yohe et al., 2018), could also shed light on whether any of the observed changes in nuclear characteristics or chromosome organization are specific to the effects of miR-206 or are associated with differentiation more generally in these cells. If the effects on nuclear and chromosome organization are mediated solely through the action of the myogenic regulatory factors, then the induction of myogenin via MEK inhibition and the potentiation of MyoD activity by increasing miR-206 levels would likely have the same effect.

Chromosomal positioning in the nucleus has been shown to be cell-type specific (Parada et al., 2004; Fritz et al., 2016), and the positioning of centromeres has been shown to change with myogenesis (Rodrigues et al., 2023), with a subset of centromeres exhibiting differential radial positioning, including chromosome 2 (Kolanowski et al., 2020). Using labeling of the entire chromosome, our data also identify differential radial positioning with myogenesis for chromosome 2, accompanied by a near doubling in the area of the nucleus the chromosome occupies. While the increased nuclear area of occupancy of chromosome 2 seen with differentiation is potentially consistent with this myogenic TGA-enriched chromosome taking on a more ‘open’ chromatin conformation to permit increased transcription of myogenic genes, we could find no evidence of the chromosome as a whole occupying an area of the nucleus with lower DNA density with differentiation. Possibilities for this finding include 1) that use of DAPI as a marker of DNA density is insufficiently sensitive to see a difference that is actually present and labeling of specific epigenetic marks (such as H3K4me3) could reveal differences, 2) that changes are present but localized to one or more portions of the chromosome and treating the chromosome as a single region for the analysis masked the difference in those areas, and/or 3) that the shift of the chromosome to a more peripheral localization, where we consistently saw higher DNA density in the DAPI staining, confounded the analysis similarly to possibility #2. More sensitive techniques, such as the use of sequencing techniques to assess DNA accessibility and contacts, could help delineate between the possibilities and relate them to regulation of the myogenic genes located on the chromosome.

The chromosome-specific differences seen in peripheral localization between the normal and RMS cells is particularly notable, especially when considered alongside the area of occupancy differences. For both chromosomes 2 and 18, RMS cells universally occupied a larger nuclear area compared to normal cells, but exhibited notably disparate behaviors in regards to localization. While chromosome 2 radial positioning was quite similar between normal and RMS cells, chromosome 18 exhibited large differences, particularly notable when examining the occupancy of the most peripheral ring, ring six. Given the association between nuclear positioning and gene regulation (Kosak et al., 2002; Ragoczy et al., 2006; Kosak et al., 2007; Robson et al., 2016), it is tempting to speculate that the enrichment for myogenic TGAs on chromosome 2 results in RMS cells continuing to appropriately regulate the positioning of that chromosome, and appropriate regulation of chromosome 18, with its lack of enrichment for myogenic TGAs, being lost. If so, we would expect it to be a more general pattern that would be observed when the radial positioning of other chromosomes is determined in both normal and RMS cells. It also suggests that analysis of gene regulation and expression in RMS cells considered on the basis of the spatial positioning of the chromosomes they reside on, rather than by clustering genes on the basis of similar function or biological processes, would demonstrate chromosome-specific differences. Others have elucidated characteristics of the organization of the genome in rhabdomyosarcoma cells using techniques including Hi-C, ChiP-Seq, and AQuA-HiChIP (Vicente-Garcia et al., 2017; Gryder et al., 2019; Wang et al., 2023). While these sequencing-based techniques have characterized aspects such as transcription factor binding, interacting genomic regions, histone marks, and regulatory elements such as enhancers, they do not directly assess chromosomal positioning or changes in such positioning, highlighting the need for dedicated analyses of spatial nuclear organization in the cells.

Our identification of differentiation-dependent preferential positioning of chromosome 2 along the major nuclear axis raises the possibility that a specific subset of chromosomes experience unique stress during myogenesis. Changes in nuclear shape–such as occurs during myogenesis–have been associated with increases in DNA damage and are related to actin activity (Pho et al., 2022). Increased DNA damage, in turn, is associated with the generation of gene-gene fusions (Seki et al., 2015). The increased frequency of chromosome 2 positioning in a specific nuclear spatial location may subject it to a different set of forces compared to a chromosome without such preferential positioning. Given that chromosome 2 is one of the chromosomes that can be involved in the characteristic PAX-FOXO gene fusions seen in a subset of RMS tumors, it suggests the possibility that the spatial positioning of chromosome 2 may contribute in some manner mechanistically to the formation of the PAX-FOXO fusions. To further test that hypothesis, the organization of other chromosomes, notably including the other chromosomes that contribute to the PAX-FOXO fusions, chromosomes 1 and 13, will need to be characterized in both models of myogenesis and rhabdomyosarcoma cells and a link established between positioning and gene fusion formation. As the other PAX gene bearing chromosome, we would anticipate chromosome 1 would have a similar preferential positioning in nuclear space to chromosome 2. Regardless of the relationship between spatial positioning of chromosome 2 and gene fusion formation, the increased alignment of chromosome 2 along the major nuclear axis with induced differentiation of the RMS cells suggests a relationship between chromosomal spatial positioning and the regulation of myogenic genes in those tumor cells, though further studies will be necessary to determine if there is a direct causal relationship.

In summary, we have identified a differentiation-dependent preferential spatial positioning of chromosome 2 in normal myocytes that is absent in both fusion-negative and fusion-positive RMS cells that is at least partially restored with induced differentiation of the tumor cells. When considered along with our data demonstrating chromosome-specific differences in other chromosomal organizational characteristics such as radial positioning, and the area of the nucleus occupied, it highlights the relevance of considering chromosomal topologies when investigating developmental processes and gene regulation in both normal and tumor cells.

## Data Availability

The datasets presented in this study can be found in online repositories. The names of the repository/repositories and accession number(s) can be found below: https://prism.northwestern.edu/badge/DOI/10.18131/r4mbp-627tcg72.svg; DOI/10.18131/7hgsf-ry967.svg; DOI/10.18131/0gvtn-chc95.svg; DOI/10.18131/x53d2-a4m37.svg; DOI/10.18131/rrz48-j6321.svg; DOI/10.18131/e1th6-bkz40.svg; and DOI/10.18131/hjan2-j5f76.svg.

## References

[B1] Begum S. Emami N. Cheung A. Wilkins O. Der S. Hamel P. A. (2005). Cell-type-specific regulation of distinct sets of gene targets by Pax3 and Pax3/FKHR. Oncogene 24 (11), 1860–1872. 10.1038/sj.onc.1208315 15688035

[B2] Bentzinger C. F. Wang Y. X. Rudnicki M. A. (2012). Building muscle: molecular regulation of myogenesis. Cold Spring Harb. Perspect. Biol. 4 (2), a008342. 10.1101/cshperspect.a008342 22300977 PMC3281568

[B3] Berkes C. A. Tapscott S. J. (2005). MyoD and the transcriptional control of myogenesis. Semin. Cell. Dev. Biol. 16 (4-5), 585–595. 10.1016/j.semcdb.2005.07.006 16099183

[B4] Boudjadi S. Pandey P. R. Chatterjee B. Nguyen T. H. Sun W. Barr F. G. (2021). A fusion transcription factor-driven cancer progresses to a fusion-independent relapse via constitutive activation of a downstream transcriptional target. Cancer Res. 81 (11), 2930–2942. 10.1158/0008-5472.CAN-20-1613 33589519 PMC8178207

[B5] Cao L. Yu Y. Bilke S. Walker R. L. Mayeenuddin L. H. Azorsa D. O. (2010). Genome-wide identification of PAX3-FKHR binding sites in rhabdomyosarcoma reveals candidate target genes important for development and cancer. Cancer Res. 70 (16), 6497–6508. 10.1158/0008-5472.CAN-10-0582 20663909 PMC2922412

[B6] Cao Y. Kumar R. M. Penn B. H. Berkes C. A. Kooperberg C. Boyer L. A. (2006). Global and gene-specific analyses show distinct roles for Myod and Myog at a common set of promoters. EMBO J. 25 (3), 502–511. 10.1038/sj.emboj.7600958 16437161 PMC1383539

[B7] Choi I. Y. Lim H. Cho H. J. Oh Y. Chou B. K. Bai H. (2020). Transcriptional landscape of myogenesis from human pluripotent stem cells reveals a key role of TWIST1 in maintenance of skeletal muscle progenitors. Elife 9, e46981. 10.7554/eLife.46981 32011235 PMC6996923

[B8] Davicioni E. Finckenstein F. G. Shahbazian V. Buckley J. D. Triche T. J. Anderson M. J. (2006). Identification of a PAX-FKHR gene expression signature that defines molecular classes and determines the prognosis of alveolar rhabdomyosarcomas. Cancer Res. 66 (14), 6936–6946. 10.1158/0008-5472.CAN-05-4578 16849537

[B9] Demsar J. Curk T. Erjavec A. Gorup C. Hocevar T. Milutinovic M. (2013). Orange: data mining toolbox in Python. J. Mach. Learn. Res. 14, 2349–2353.

[B10] Doynova M. D. Markworth J. F. Cameron-Smith D. Vickers M. H. O'Sullivan J. M. (2017). Linkages between changes in the 3D organization of the genome and transcription during myotube differentiation *in vitro* . Skelet. Muscle 7 (1), 5. 10.1186/s13395-017-0122-1 28381300 PMC5382473

[B11] Fritz A. J. Barutcu A. R. Martin-Buley L. van Wijnen A. J. Zaidi S. K. Imbalzano A. N. (2016). Chromosomes at work: organization of chromosome territories in the interphase nucleus. J. Cell. Biochem. 117 (1), 9–19. 10.1002/jcb.25280 26192137 PMC4715719

[B12] Gryder B. E. Pomella S. Sayers C. Wu X. S. Song Y. Chiarella A. M. (2019). Histone hyperacetylation disrupts core gene regulatory architecture in rhabdomyosarcoma. Nat. Genet. 51 (12), 1714–1722. 10.1038/s41588-019-0534-4 31784732 PMC6886578

[B13] Gryder B. E. Yohe M. E. Chou H. C. Zhang X. Marques J. Wachtel M. (2017). PAX3-FOXO1 establishes myogenic super enhancers and confers BET bromodomain vulnerability. Cancer Discov. 7 (8), 884–899. 10.1158/2159-8290.CD-16-1297 28446439 PMC7802885

[B14] Hanna J. A. Garcia M. R. Lardennois A. Leavey P. J. Maglic D. Fagnan A. (2018). PAX3-FOXO1 drives miR-486-5p and represses miR-221 contributing to pathogenesis of alveolar rhabdomyosarcoma. Oncogene 37 (15), 1991–2007. 10.1038/s41388-017-0081-3 29367756 PMC5895609

[B15] Hibbitts E. Chi Y. Y. Hawkins D. S. Barr F. G. Bradley J. A. Dasgupta R. (2019). Refinement of risk stratification for childhood rhabdomyosarcoma using FOXO1 fusion status in addition to established clinical outcome predictors: a report from the Children's Oncology Group. Cancer Med. 8 (14), 6437–6448. 10.1002/cam4.2504 31456361 PMC6797586

[B16] Hinson A. R. Jones R. Crose L. E. Belyea B. C. Barr F. G. Linardic C. M. (2013). Human rhabdomyosarcoma cell lines for rhabdomyosarcoma research: utility and pitfalls. Front. Oncol. 3, 183. 10.3389/fonc.2013.00183 23882450 PMC3713458

[B17] Ibarra J. Hershenhouse T. Almassalha L. M. MacQuarrie K. L. (2023). Differentiation-dependent chromosomal organization changes in normal myogenic cells are absent in rhabdomyosarcoma cells. bioRxiv, 2023.05.11.540394. 10.1101/2023.05.11.540394 PMC10662331 38020905

[B18] Kolanowski T. J. Rozwadowska N. Zimna A. Nowaczyk M. Siatkowski M. Labedz W. (2020). Chromatin and transcriptome changes in human myoblasts show spatio-temporal correlations and demonstrate DPP4 inhibition in differentiated myotubes. Sci. Rep. 10 (1), 14336. 10.1038/s41598-020-70756-x 32868771 PMC7459101

[B19] Kosak S. T. Scalzo D. Alworth S. V. Li F. Palmer S. Enver T. (2007). Coordinate gene regulation during hematopoiesis is related to genomic organization. PLoS Biol. 5 (11), e309. 10.1371/journal.pbio.0050309 18031200 PMC2080650

[B20] Kosak S. T. Skok J. A. Medina K. L. Riblet R. Le Beau M. M. Fisher A. G. (2002). Subnuclear compartmentalization of immunoglobulin loci during lymphocyte development. Science 296 (5565), 158–162. 10.1126/science.1068768 11935030

[B21] MacQuarrie K. L. Yao Z. Fong A. P. Diede S. J. Rudzinski E. R. Hawkins D. S. (2013). Comparison of genome-wide binding of MyoD in normal human myogenic cells and rhabdomyosarcomas identifies regional and local suppression of promyogenic transcription factors. Mol. Cell. Biol. 33 (4), 773–784. 10.1128/MCB.00916-12 23230269 PMC3571334

[B22] Macquarrie K. L. Yao Z. Young J. M. Cao Y. Tapscott S. J. (2012). miR-206 integrates multiple components of differentiation pathways to control the transition from growth to differentiation in rhabdomyosarcoma cells. Skelet. Muscle 2 (1), 7. 10.1186/2044-5040-2-7 22541669 PMC3417070

[B23] McEvoy M. T. Siegel D. A. Dai S. Okcu M. F. Zobeck M. Venkatramani R. (2023). Pediatric rhabdomyosarcoma incidence and survival in the United States: an assessment of 5656 cases, 2001-2017. Cancer Med. 12 (3), 3644–3656. 10.1002/cam4.5211 36069287 PMC9939205

[B24] Neems D. S. Garza-Gongora A. G. Smith E. D. Kosak S. T. (2016). Topologically associated domains enriched for lineage-specific genes reveal expression-dependent nuclear topologies during myogenesis. Proc. Natl. Acad. Sci. U. S. A. 113 (12), E1691–E1700. 10.1073/pnas.1521826113 26957603 PMC4812766

[B25] Parada L. A. McQueen P. G. Misteli T. (2004). Tissue-specific spatial organization of genomes. Genome Biol. 5 (7), R44. 10.1186/gb-2004-5-7-r44 15239829 PMC463291

[B26] Penn B. H. Bergstrom D. A. Dilworth F. J. Bengal E. Tapscott S. J. (2004). A MyoD-generated feed-forward circuit temporally patterns gene expression during skeletal muscle differentiation. Genes. Dev. 18 (19), 2348–2353. 10.1101/gad.1234304 15466486 PMC522984

[B27] Pho M. Berrada Y. Gunda A. Lavallee A. Chiu K. Padam A. (2022). Actin contraction controls nuclear blebbing and rupture independent of actin confinement. bioRxiv. 2022.2012.2001.518663. 10.1091/mbc.E23-07-0292 PMC10881147 38088876

[B28] Ragoczy T. Bender M. A. Telling A. Byron R. Groudine M. (2006). The locus control region is required for association of the murine beta-globin locus with engaged transcription factories during erythroid maturation. Genes. Dev. 20 (11), 1447–1457. 10.1101/gad.1419506 16705039 PMC1475758

[B29] Rao P. K. Missiaglia E. Shields L. Hyde G. Yuan B. Shepherd C. J. (2010). Distinct roles for miR-1 and miR-133a in the proliferation and differentiation of rhabdomyosarcoma cells. FASEB J. 24 (9), 3427–3437. 10.1096/fj.09-150698 20466878 PMC3231107

[B30] Robson M. I. de Las Heras J. I. Czapiewski R. Le Thanh P. Booth D. G. Kelly D. A. (2016). Tissue-specific gene repositioning by muscle nuclear membrane proteins enhances repression of critical developmental genes during myogenesis. Mol. Cell. 62 (6), 834–847. 10.1016/j.molcel.2016.04.035 27264872 PMC4914829

[B31] Rodrigues A. MacQuarrie K. L. Freeman E. Lin A. Willis A. B. Xu Z. (2023). Nucleoli and the nucleoli-centromere association are dynamic during normal development and in cancer. Mol. Biol. Cell. 34 (4), br5. br5. 10.1091/mbc.E22-06-0237 36753381 PMC10092642

[B32] Rozwadowska N. Kolanowski T. Wiland E. Siatkowski M. Pawlak P. Malcher A. (2013). Characterisation of nuclear architectural alterations during *in vitro* differentiation of human stem cells of myogenic origin. PLoS One 8 (9), e73231. 10.1371/journal.pone.0073231 24019912 PMC3760906

[B33] Rudzinski E. R. Kelsey A. Vokuhl C. Linardic C. M. Shipley J. Hettmer S. (2021). Pathology of childhood rhabdomyosarcoma: a consensus opinion document from the children's oncology group, European paediatric soft tissue sarcoma study group, and the cooperative weichteilsarkom studiengruppe. Pediatr. Blood Cancer 68 (3), e28798. 10.1002/pbc.28798 33306276

[B34] Schindelin J. Arganda-Carreras I. Frise E. Kaynig V. Longair M. Pietzsch T. (2012). Fiji: an open-source platform for biological-image analysis. Nat. Methods 9 (7), 676–682. 10.1038/nmeth.2019 22743772 PMC3855844

[B35] Seki Y. Mizukami T. Kohno T. (2015). Molecular process producing oncogene fusion in lung cancer cells by illegitimate repair of DNA double-strand breaks. Biomolecules 5 (4), 2464–2476. 10.3390/biom5042464 26437441 PMC4693243

[B36] Stirling D. R. Swain-Bowden M. J. Lucas A. M. Carpenter A. E. Cimini B. A. Goodman A. (2021). CellProfiler 4: improvements in speed, utility and usability. BMC Bioinforma. 22 (1), 433. 10.1186/s12859-021-04344-9 PMC8431850 34507520

[B37] Sunkel B. D. Wang M. LaHaye S. Kelly B. J. Fitch J. R. Barr F. G. (2021). Evidence of pioneer factor activity of an oncogenic fusion transcription factor. iScience 24 (8), 102867. 10.1016/j.isci.2021.102867 34386729 PMC8346656

[B38] Tapscott S. J. Thayer M. J. Weintraub H. (1993). Deficiency in rhabdomyosarcomas of a factor required for MyoD activity and myogenesis. Science 259 (5100), 1450–1453. 10.1126/science.8383879 8383879

[B39] Taulli R. Bersani F. Foglizzo V. Linari A. Vigna E. Ladanyi M. (2009). The muscle-specific microRNA miR-206 blocks human rhabdomyosarcoma growth in xenotransplanted mice by promoting myogenic differentiation. J. Clin. Investig. 119 (8), 2366–2378. 10.1172/JCI38075 19620785 PMC2719932

[B40] Vicente-Garcia C. Villarejo-Balcells B. Irastorza-Azcarate I. Naranjo S. Acemel R. D. Tena J. J. (2017). Regulatory landscape fusion in rhabdomyosarcoma through interactions between the PAX3 promoter and FOXO1 regulatory elements. Genome Biol. 18 (1), 106. 10.1186/s13059-017-1225-z 28615069 PMC5470208

[B41] Wang M. Sreenivas P. Sunkel B. D. Wang L. Ignatius M. Stanton B. Z. (2023). The 3D chromatin landscape of rhabdomyosarcoma. Nar. Cancer 5 (3), zcad028. 10.1093/narcan/zcad028 37325549 PMC10261698

[B42] Wolfram Research I. (2022). Mathematica.Version 13.2.

[B43] Yang Z. MacQuarrie K. L. Analau E. Tyler A. E. Dilworth F. J. Cao Y. (2009). MyoD and E-protein heterodimers switch rhabdomyosarcoma cells from an arrested myoblast phase to a differentiated state. Genes. Dev. 23 (6), 694–707. 10.1101/gad.1765109 19299559 PMC2661613

[B44] Yohe M. E. Gryder B. E. Shern J. F. Song Y. K. Chou H. C. Sindiri S. (2018). MEK inhibition induces MYOG and remodels super-enhancers in RAS-driven rhabdomyosarcoma. Sci. Transl. Med. 10 (448), eaan4470. 10.1126/scitranslmed.aan4470 29973406 PMC8054766

[B45] Zhang S. Wang J. Liu Q. McDonald W. H. Bomber M. L. Layden H. M. (2022). PAX3-FOXO1 coordinates enhancer architecture, eRNA transcription, and RNA polymerase pause release at select gene targets. Mol. Cell. 82 (23), 4428–4442.e7. 10.1016/j.molcel.2022.10.025 36395771 PMC9731406

