# Supplementary figures and images for: Differentiation-dependent chromosomal organization changes in normal myogenic cells are absent in rhabdomyosarcoma cells
Source: Front Cell Dev Biol. 2023 Nov 7;11:1293891. doi: 10.3389/fcell.2023.1293891 (PMC10662331; doi:10.3389/fcell.2023.1293891)

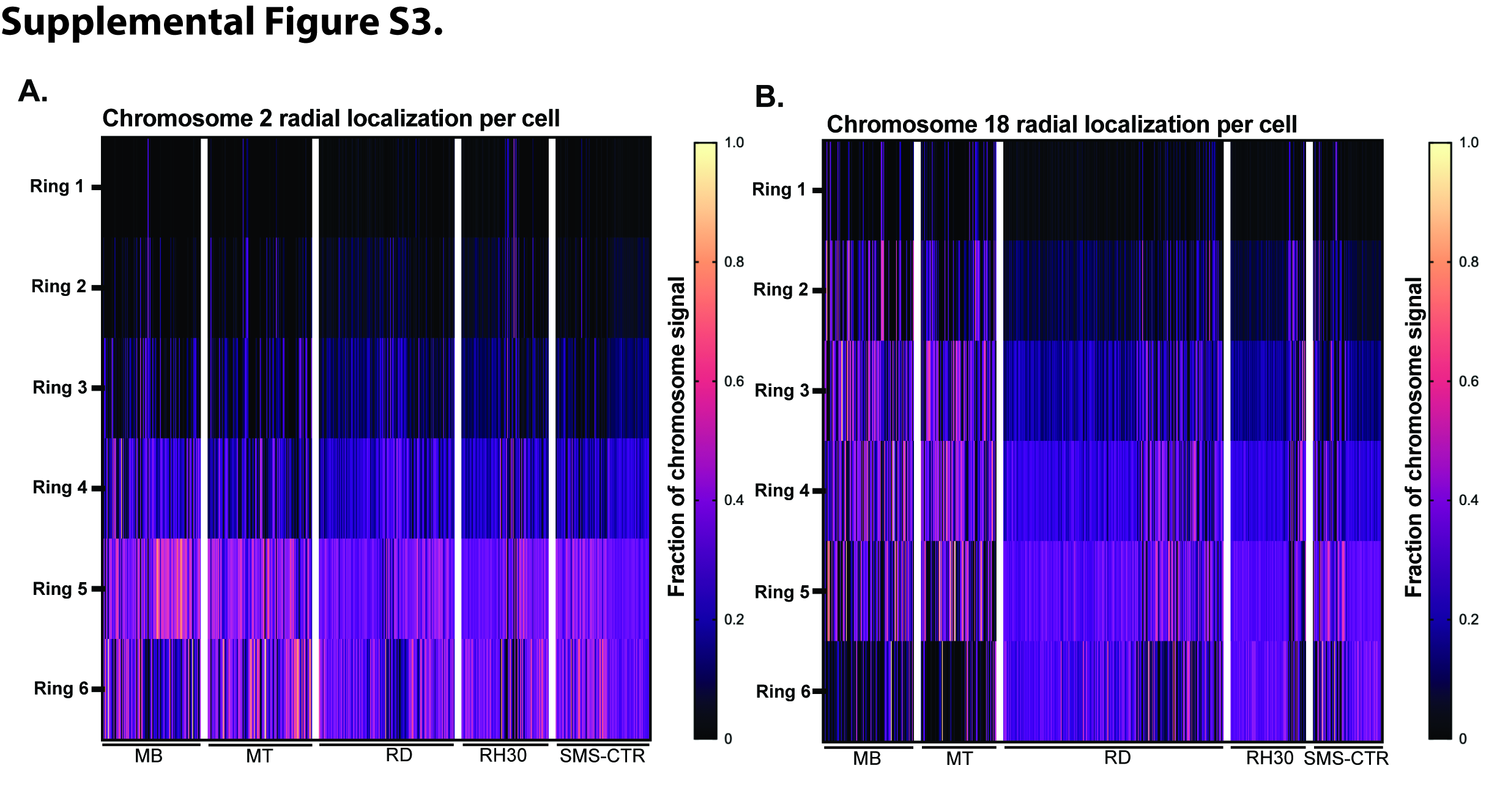

Supplement: Supplementary file 2 [file Image3.TIF]

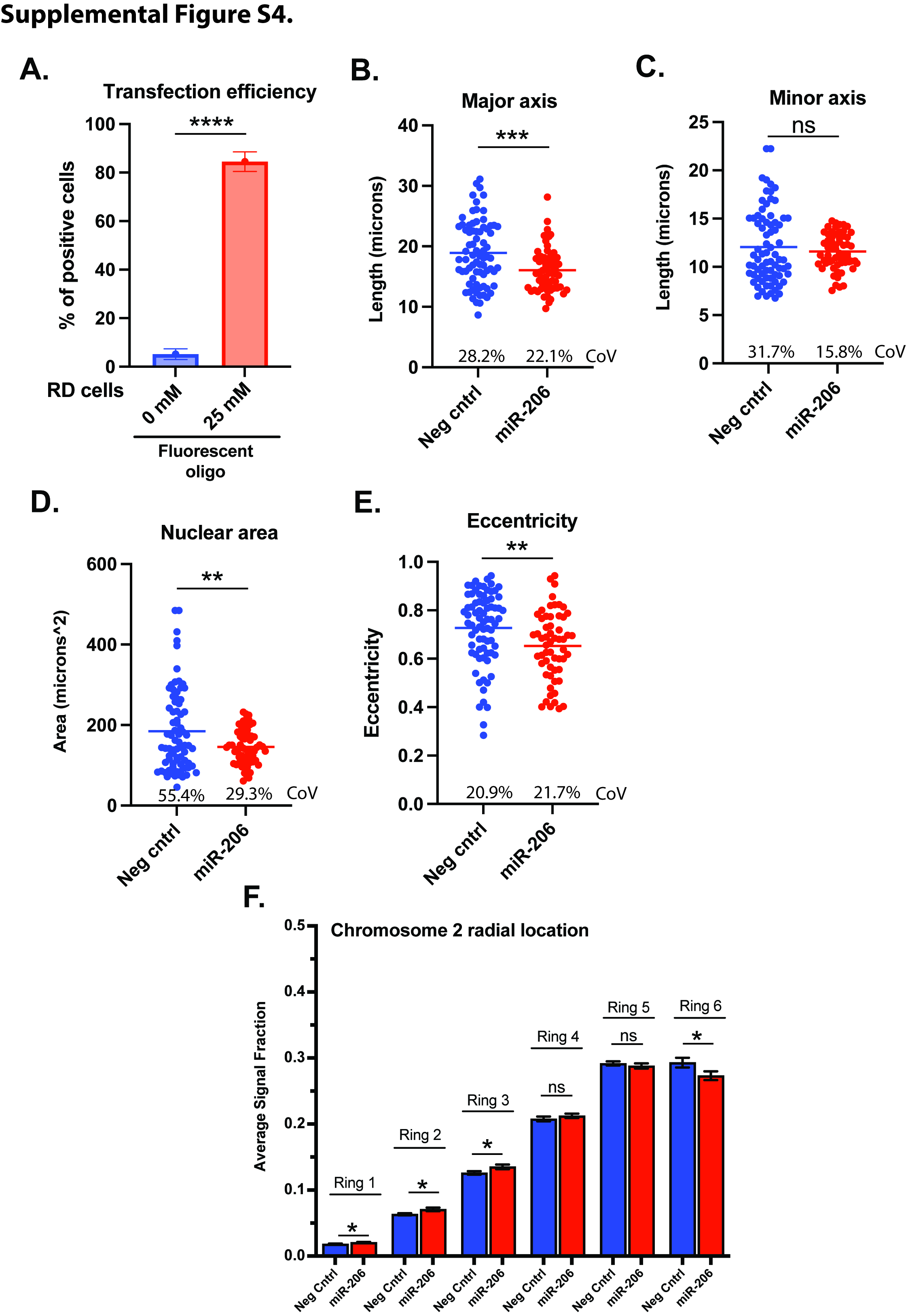

Supplement: Supplementary file 3 [file Image4.TIF]

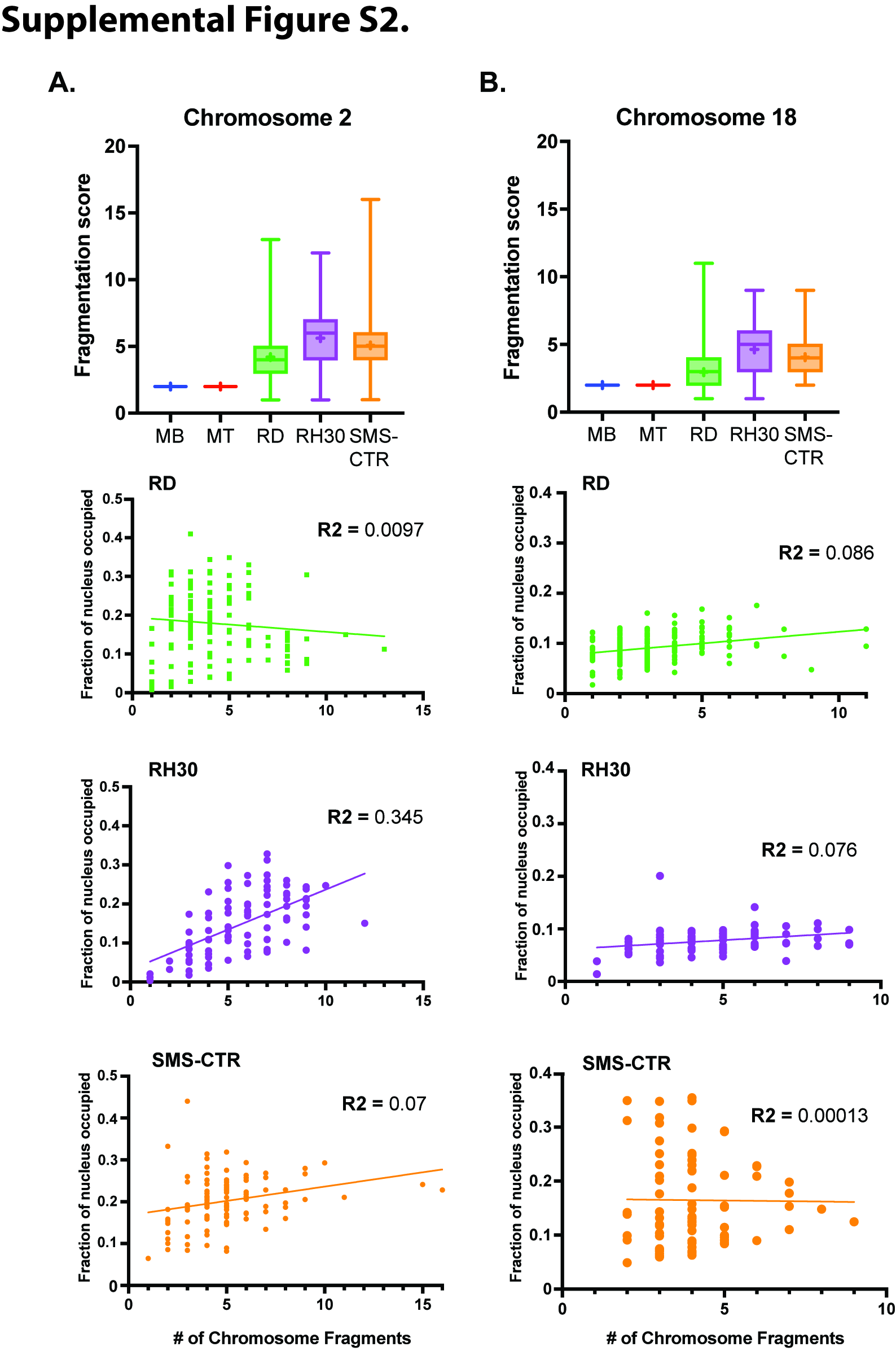

Supplement: Supplementary file 4 [file Image2.TIF]

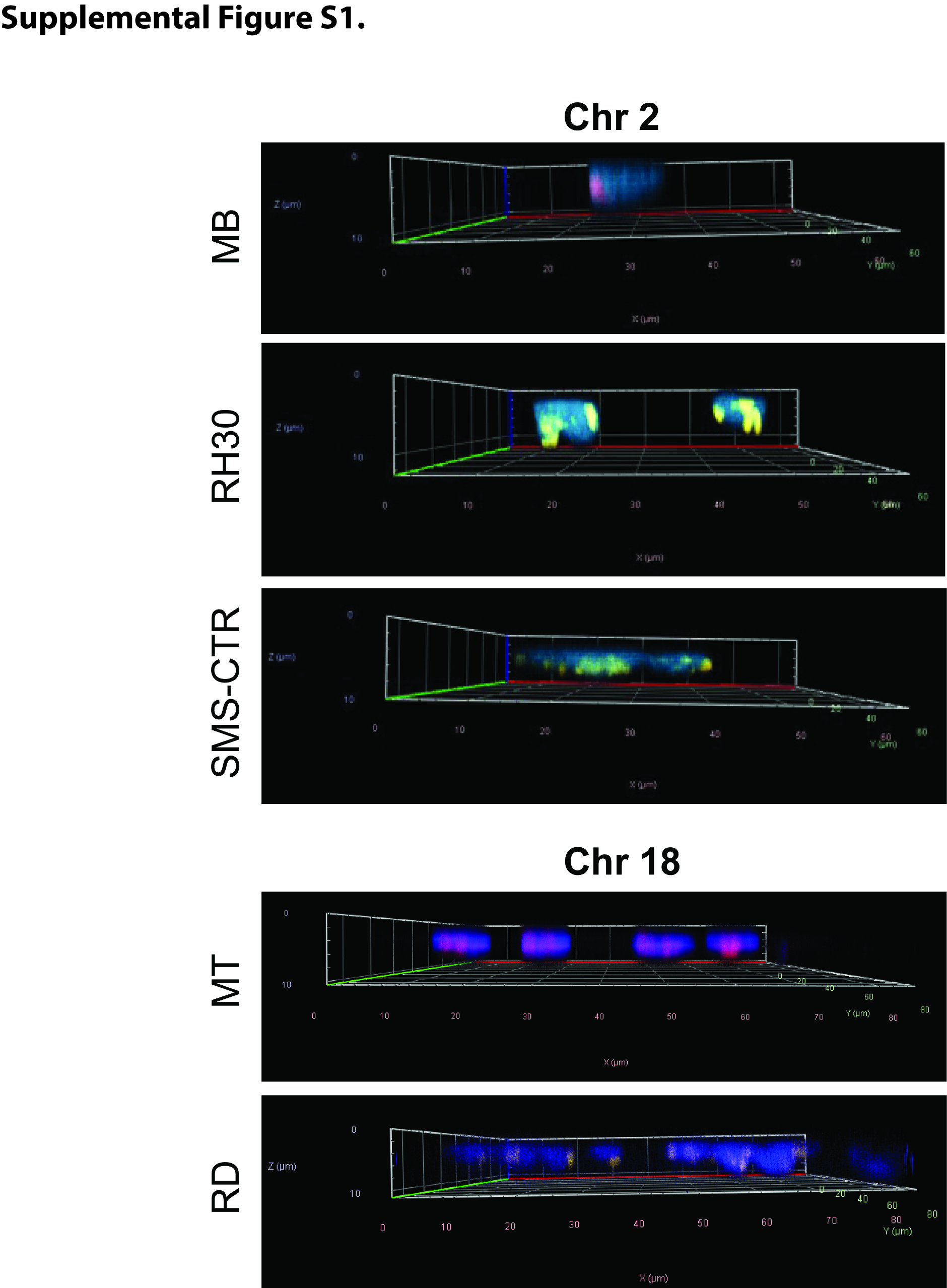

Supplement: Supplementary file 5 [file Image1.TIF]
