# Supplementary material for: Differentiation-dependent chromosomal organization changes in normal myogenic cells are absent in rhabdomyosarcoma cells
Source: Front Cell Dev Biol. 2023 Nov 7;11:1293891. doi: 10.3389/fcell.2023.1293891 (PMC10662331; doi:10.3389/fcell.2023.1293891)
Supplement: Supplementary file 7 [file DataSheet1.docx]

**Supplemental Figure Legends**

**Supplemental Figure S1. Chromosomes 2 and 18 occupy the majority of the z-axis in individual cells.** Representative reconstructions of chromosome signal in the z-axis are shown for chromosomes 2 and 18 in cell types as indicated.

**Supplemental Figure S2. Chromosomal nuclear area occupancy is not a function of chromosome fragmentation.** A) The chromosome fragmentation score (ie. number of detected distinct areas of chromosome paint per nucleus) is shown as a bar and whiskers plot (horizontal line in box: median, ‘+’: mean, whiskers indicate minimum and maximum observed values) for each cell type from the same cells analyzed in Figure 2. As expected, all myoblast (MB) and myotube (MT) nuclei exhibited 2 countable areas of signal per cell, while the tumor cell lines showed variability. Scatterplots below show the fraction of the nucleus occupied per nucleus graphed as a function of the computed fragmentation score for each of the 3 tumor cell lines and a computed regression line. Least squares regression for each cell type showed a negative correlation for RDs, positive correlations for RH30s and SMS-CTRs, and low R2 values overall (as indicated on each graph). B) Chromosome fragmentation scores for chromosome 18 as in S2A, again showing only 2 countable areas of chromosome signal per nucleus for the normal cells, and variable numbers for the tumor cell lines. As for chromosome 2, plots of occupied nuclear area as a function of fragmentation score for each cell type again show significant variability, and very low R2 values with least squares regression. n = 93 – 110 nuclei per cell type.

**Supplemental Figure S3. The pattern of chromosome radial localization in individual cells is similar to what is seen in average measurements.** Heatmaps for the chromosome radial localization of chromosomes 2 and 18 shown for the individual cells that were averaged in Figures 2E and 2F. Each column represents the data from an individual nucleus.

**Supplemental Figure S4. miR-206 transfection in RD cells, nuclear characteristics, and chromosome 2 radial location.** A) RD cells can be transfected at a high rate of efficiency. Transfection of a fluorescently labeled oligo at the concentration indicated results in >80% of cells being scored as positive for signal. Fewer than 5% of control cells (no oligo) are scored as positive using the same criteria. B) The major axis of miR-206 mimetic transfected RD cells are slightly shorter on average compared to cells transfected with a negative control mimetic and have a smaller coefficient of variation (CoV). C) The minor axis of miR-206 transfected cells is not significantly different on average compared to control transfected cells but has a smaller CoV. D) The nuclear area of miR-206 transfected cells is smaller on average compared to control cells, as well as having a smaller CoV. E) miR-206 cells are less eccentric on average, and have a similar CoV to negative control cells. F) Average radial localization of chromosome 2 was computed as a function of six concentric nuclear rings as in Figure 2. All statistical tests were performed as t-tests with unequal variance. ns: not significant; *: p<0.05; **: p<0.01; ***: p<0.001; ****: p<0.0001; n = 76 and 61 nuclei for negative control and miR-206 nuclei, respectively.
